# Valuing Changes in Time Use in Low- and Middle-Income Countries

**DOI:** 10.1017/bca.2018.21

**Published:** 2018-12-19

**Authors:** Dale Whittington, Joseph Cook

**Affiliations:** 1 Departments of Environmental Sciences & Engineering and City & Regional Planning, University of North Carolina at Chapel Hill, USA; 2 Global Development Institute, School of Environment, Education, and Development, University of Manchester, UK; 3 School of Economic Sciences, Washington State University, Pullman, WA, USA

**Keywords:** benefits transfer, development, environment, health, international, transportation, value of time

## Abstract

Valuing changes in time use is often a critical element of economic analyses of development projects. In this paper we review the literature on the monetary value of time in low- and middle-income countries and find support for a commonly used benchmark of 50% of after-tax wages for time changes in activities in the informal sector, such as collecting water or traveling to health clinics. We offer recommendations to analysts who are conducting benefit-cost analyses in these settings about what methods they can use to estimate the value of time. These include a benefits transfer approach and also a relatively simple stated preference approach that might be deployed in a specific context if the project recommendation is sensitive to the assumption of the value of time or if the distribution of the benefits of time savings is especially important.

**JEL classifications:** J22; O22.

## 1 Introduction

One important outcome of some development projects in low- and middle-income countries is that households may no longer need to spend as much time to accomplish a specific activity, and can thus devote these “time savings” to other welfare-enhancing activities. For example, a health clinic may be located closer to a household’s community, and thus members of the household will have to spend less time traveling to receive both routine and emergency health services. An improved water source may be located closer to a household, and thus women and children who collect water will need to spend less time to collect a given quantity of water. In both examples, households experience reductions in travel times. Households may choose to spend these travel time savings on other pursuits, such as leisure or paid labor, or they may use the travel time savings to travel more frequently (i.e., visit the health clinic more often or collect more water).

Changes in households’ time allocation also can be negative (a cost to the household). A side effect of a health or development policy or project may be that households have to spend more time on a specific activity, and thus incur a welfare loss. For example, a road project may displace some households, forcing them to relocate farther away from their work or school. As a consequence, they may spend more time commuting. In this case the change in time allocation would decrease individuals’ well-being and should be counted as a cost of the policy intervention.

Having a health clinic or an improved water source closer to one’s home is an economic benefit, and a common approach to measuring the magnitude of this benefit is to multiply the amount of time “saved” in the activity by a monetary value per time unit (shadow value of time). The purpose of this paper is to summarize the literature on the monetary value of time in developing countries and to offer recommendations to analysts who are conducting benefit-cost analyses in low- and middle-income countries about what methods they can use to estimate the value of time. If conducting primary research on the value of changes in time use is not feasible or warranted, we suggest a parameter value they might use in a benefit-cost analysis instead.

This parameter value is commonly referred to as the “value of time” (VOT). This name implies that there is a single value for an individual or household that characterizes their opportunity cost of time. It is more accurate, however, to refer to the value of time saved in specific activities like collecting water, traveling to health clinics, or waiting at a government office. Assuming similar VOT estimates across these sectors implies that marginal utility of time spent waiting in an office, walking to a vaccination clinic, or collecting water from a source outside the home, are similar. This seems implausible. Researchers in the transportation sector, where VOT is typically referred to as the “value of travel time” (VTT) or “value of travel time savings” (VTTS), have long recognized that different values should be used, for example, for time spent in free-flow traffic vs. congested traffic or for travel with different modes (US Department of Transportation, 2016). Therefore, one should not expect VOT estimates to be easily transferable from one sector to another. Furthermore, these values are likely to vary among individuals even for the same activities. For example, one person may find waiting in line to be vaccinated drudgery while another enjoys the time socializing with community members. Since the marginal utility of waiting varies between individuals, so too should their value of changes in the queuing time (these might occur, for example, by adding vaccination staff and lowering wait times).

When time changes occur in the activities of employees, there is a consensus among economists working in industrialized countries that before-tax market wage data should be used to estimate the value of time, including benefits and indirect costs of employee supervision since this is the full opportunity cost to society of the employees’ time (Robinson et al., 2017; Baxter et al., 2017; US HHS, 2016).^1^ This conclusion would hold in developing countries if the development project affected the time use of salaried employees on the job who paid taxes and received benefits.

However, it is more common in developing countries than in industrialized countries for people to be working outside the formal sector and not be paying taxes. In developing countries many people, especially women, do not have jobs in the formal sector. Changes in their time allocation occur in the informal economy and the home, and valuing these changes in time allocation requires nonmarket valuation approaches. When time changes occur in a household’s activities outside of salaried employment, the value may be more uncertain. Economists in industrialized countries often use the after-tax wage rate of the individual(s) affected to approximate this value, since this is the relevant opportunity cost faced by the individual (Baxter et al., 2017). This conclusion holds if the individual has the opportunity to tradeoff (exchange) time in nonmarket activities with activities in the formal sector.^2^

In both industrialized and developing countries, individuals who save time in household or informal activities may have opportunities to reallocate time savings to activities that generate income. But for a variety of reasons, this may not be the case. For example, a project or policy intervention may reduce or increase an individual’s commuting time without affecting the amount of time the individual is required to spend at a salaried job. However, individuals may impute a value to time savings even in the absence of income-generation opportunities, but this may not be equal to their after-tax wage rate. And even if income-generating activities are available, individuals may decide to devote time savings to other nonmarket activities such as childcare or leisure, and it is an empirical question what economic value individuals assign to time savings devoted to activities that do not directly generate additional monetary income.

Nonmarket valuation approaches are also required to estimate the value of time in both industrialized countries and developing countries when policy interventions affect time use patterns outside an employee’s normal work day. Just as in developing countries, many people in industrialized countries work at home or outside the formal sector. However, in developing countries nonmarket valuation techniques play a larger role in estimates of the value of changes in time use because few people are employed in the formal sector.

Economists in industrialized countries have thus used nonmarket valuation methods (both revealed and stated preference techniques) to estimate the value of time changes that do not occur under an employer’s watch, predominantly time spent traveling. Economists have interpreted the results of this research in industrialized countries to suggest that a reasonable estimate of the VTT savings would be 50% of an individual’s after-tax wages (Boardman et al., 2018; von Wartburg & Waters, 2004). These authors also recommend valuing travel time savings from walking at double this amount (100% of wages), and waiting at 125% of wages.^3^ But there has been little discussion in the development economics literature as to whether these conclusions are applicable in developing countries.

This paper is organized in five sections. The next, second section reviews the literature on the value of changes in time use in low- and middle-income countries. In the third section, we describe a benefit transfer approach to estimating the value of time changes in low- and middle-income countries. The fourth section describes a stated preference approach that can be used to estimate the value of time for a specific development project (i.e., an estimate for a specific local context where households’ time use patterns will be affected by a development project). In the fifth section, we present our recommendations on what a benefit-cost analyst should do if one of the outcomes of a development project being appraised in a low- or middle-income country is a change in households’ time use patterns.

## 2 Literature review

There are relatively few empirical studies of the value of time savings in low- and middle-income countries. Table 1 presents a summary of eleven papers that we reviewed. As shown, this literature is quite recent. All but two of these studies have been published in the last 10 years. Six of the studies are from countries in Africa (three of these from Kenya), four from Asia (two of these are from China), and one from Latin America (Costa Rica). Five of the studies used revealed preference methods, four used stated preference methods, and two used both. Four of the studies report value of time estimates for the transport sector (value of time spent commuting), and four report estimates for the water supply sector (value of time spent traveling and waiting to collect water). There is one study examining travel times in the context of the health sector. Only two of the studies examine time spent outside the context of travel: one for the labor sector, and one for reductions in wait times for a generic public service. Six of the studies were conducted in large urban areas, four in rural areas, and one in a small town.

**Table 1:** A summary of the literature on estimates of the value of time (vot) in low- and middle-income countries.

Author(s)	Sector	Country	Location	Valuation method	Date of fieldwork	Sample size	Results	Comments
Whittington et al. (1990)	Water supply	Kenya	Ukunda (small market town)	Revealed preference (actual water source choices, MNL)	1986	69	“Bounding” results (pp. 273–274) imply VTT ~100% imputed wages; RUM results imply VTT ~125% of local unskilled wages	
Asthana (1997)	Water supply	India	Rural	Revealed preferences (actual water source choices, probit)	?	245	VTT ~ 35% of the unskilled wage rate (as fixed by Labor Commission)	Little information on survey effort or sample frame
Dissanayake and Morikawa (2002)	Transport	Thailand	Bangkok	Revealed preference (actual mode choice, nested logit)	1995	1,205	Mean VTT of 27 Thai baht/hour, but no description of average wage rates in sample. Cost parameter expressed as fraction of income	First level of nested model is ownership of car or motorcycle; lower level models mode choice. Two-commuter households only
Alpizar and Carlsson (2003)	Transport	Costa Rica	San Jose	Stated preference (repeated discrete choice, MNL and RPL)	2000	602	Mean values of VTT of 40%–50% of the sample’s average hourly wages, but sensitive to econometric specification	Frame limited to current car owners
Liu (2007)	Transport	China	Shanghai	Revealed & stated preference (actual mode choice & contingent valuation)	2001	100 (useable sample of 91)	VTT estimates averaged 64% of in-sample wage rates for in-vehicle time and 82% of wages for out-of-vehicle time	No information on sampling strategy or representativeness
Walker et al. (2010)	Transport	China	Chengdu	Revealed & stated preference (actual mode choice & contingentvaluation)	2005	532 commute trip choices from 1,001 sampled households	Average VTT 7.8–12.9 yuan per hour, 51%–86% of city-wide average income	
Jeuland et al. (2010)	Health	Mozambique	Beira	Revealed preference (travel cost)	2005	1300	Estimated the VTT 18%–46% of the median hourly wage in sample	Household travel cost model of decision to participate in a vaccine trial; did not distinguish between utility of traveling and queuing
Kremer et al. (2011)	Water supply	Kenya	Rural	Stated preference (double-bounded, dichotomous choice contingent valuation)	2005	104	$0.09 per 8-hour day; 7% of unskilled or casual labor wage rate	Willingness to pay for protected springs asked as separate exercise from “willingness to walk.” VTT as ratio of these two (n D 104); estimate reported is for an out of sample prediction to other study participants
Larson et al. (2016)	Labor market choices	Botswana	Rural	Stated preference (contingent behavior)	2007	499 households in 13 villages	VTT varies by job characteristics but average BWP 8–12 per day for men, 17–21 for women	Willingness to accept wildlife conservation jobs; job type, daily wage, number of days worked per month, and job duration varied. Model allowing VTT to vary with money income, time available, wage offered and days worked fit data better than constant VTT parameter
Wondemu (2016)	Waiting for public services	South Africa, Nigeria, Ethiopia	Johannesburg, Addis Ababa, Lagos	Stated preference (open-ended max WTP)	2011	1,296 in total	69%, 66%, and 74% of city-wide average wage rate in Addis Ababa, Jo’burg and Lagos, respectively	Surveyed only those currently employed; little detail on survey sampling frame
Cook et al. (2016a,b)	Water supply	Kenya	Rural (Meru)	Stated preference (discrete choice experiment, RPL and latent class)	2013	387 in four “sublocations”	50% of the household’s wage rate but heterogeneity	Among households without private piped connections

We follow the convention in the transportation and recreation demand literatures, and report VOT estimates as a fraction of hourly wage rates if authors report this. As we discuss below, this wage rate could be directly observed through surveys or from secondary data, and calculated either as the hourly wage (for hourly workers) or imputed based on annual income and an assumption about the number of working hours per year. Another common approach is to report VOT estimates as a fraction of local unskilled wage rates. Unfortunately, none of the eleven studies distinguishes between before- and after-tax wages. It seems most likely to us that in most cases what was observed was after-tax wages because the informal economy is larger in these settings and fewer workers would be earning taxable wages.

In the transportation sector, Dissanayake and Morikawa (2002) and Walker et al. (2010) estimated the VTT by examining mode (bus, car, train) choices in a revealed preference, nested logit framework. Dissanayake and Morikawa (2002) report a mean VTT of 27 baht per hour in Bangkok in 1995. The authors do not report results as a fraction of wages or income. Walker et al. (2010) report a range of VTT in Chengdu, China of 51%–86% of the city-wide average hourly wages.^4^

Liu (2007) used both actual mode choice and data from stated preference questions to rank-order respondents’ transportation choices. VTT estimates averaged 64% of in-sample wage rates for in-vehicle time and 82% of wages for out-of-vehicle time.

Alpizar and Carlsson (2003) used a repeated discrete choice approach, asking car commuters in San Jose, Costa Rica to make several hypothetical choices between continuing to commute by car or switching to a public bus. The authors model these data using a random-parameters logit framework, and find mean values of VTT of 40%–50% of the sample’s hourly wages. Respondents were willing to pay more for reductions in travel time by bus than by car.

Jeuland et al. (2010) applied the travel cost method to individuals’ decisions to travel and queue to receive free cholera vaccines in Beira, Mozambique. Using a count model of visits from surveyed households throughout the city, and survey data on the pecuniary cost of travel (i.e., bus fares) and respondents’ total travel times, they estimated respondents’ VTT as 18%–46% of the median hourly wage.

Larson et al. (2016) used a contingent behavior approach and asked respondents in rural Botswana to make a series of choices among hypothetical jobs in community-based natural resource programs.^5^ Each choice task offered a respondent different job offers that differed by type of activity, daily wage, and days of month to be worked. The authors used a flexible model of labor decisions in which the shadow value of time varies with a number of economic variables rather than implying a single, constant value of time. The authors used the model results to estimate the minimum wage respondents would accept for each job type.

Wondemu (2016) surveyed a large sample of employed residents in Lagos (Nigeria), Johannesburg (South Africa) and Addis Ababa (Ethiopia) and asked their maximum willingness to pay to reduce waiting times for a generic public service. The implied WTP to reduce waits by one hour are 69%, 66%, and 74% of city-wide average wage rates in the three cities. However, the paper uses an open-ended elicitation question which is not incentive-compatible and thus not recommended in state-of-the-art stated preference studies (Johnston et al., 2017). Given the sampling strategy, the wage fractions are also relevant only to those who are currently employed.

Whittington et al. (1990) used two revealed preference methods to estimate the VTT spent collecting water from outside the home in a small market town (Ukunda) in eastern Kenya. Both methods relied on actual water source decisions. The first approach bounded VTTs by exploiting differences in collection times (including walking, waiting and filling containers) and prevailing prices paid between free open wells, water kiosks, and water vendors who would deliver water to the house, along with times needed to collect water from each of these. The second used a multinomial logit discrete choice framework. Both approaches used data from 69 households and found that the VTT was approximately 100% of unskilled wages.

Asthana (1997) analyzed water source choice decisions of 490 households in rural India using a discrete choice model. The author estimated the VTT to be approximately 35% of the unskilled wage rate.

Kremer et al. (2011) examined individuals’ decisions to travel to springs that had been randomly selected for protection from water quality contamination in rural Kenya. Actual water source choices, as well as stated rankings of sources, were modeled with a random-parameters logit framework. The authors’ main research objective was to estimate how households valued improvements in water quality. To estimate the value of time spent collecting water, however, the authors used stated preference data from a double-bounded, dichotomous choice contingent valuation task. In the first step of the valuation task, respondents were asked how much their household would be willing to pay to “keep their spring protected.” In the second step, respondents were asked how many minutes their household would be willing to walk to obtain water from a protected spring. For the 104 respondents with answers to both questions, the authors divided the willingness to walk by the willingness to pay to derive the VTT. Their estimate has a mean of US$0.09 per 8-hour day, or only 7% of unskilled or casual labor wage rates.^6^

Cook et al. (2016*a*) also used stated preference data to estimate the value of time spent collecting water in rural Kenya, but their approach differs from Kremer et al. (2011). Their valuation task explicitly presents respondents with the tradeoff between time and money, rather than relying on the ratio of two separate valuation exercises. This approach also allowed Cook et al. (2016*a*,*b*) to model responses in a random-parameters logit framework and to report individual-level VTT estimates. Also, in the Kremer et al. (2011) study, the average self-reported one-way walking time in their site was only 9 minutes so that time spent collecting water may have been less salient to respondents than in the study site in Cook et al. (2016*a*), where average one-way walk times were 22 minutes.

Cook et al. found that there was considerable heterogeneity in the value of time savings among their sample households. A latent class modeling approach revealed four categories of respondents. About a third of sample respondents valued the time savings quite highly – at 140% of the prevailing unskilled wage rates. For these households, time savings were clearly valuable, either because they could be reallocated to income-generating activities, or because the household valued the fact that more time could be spent on other non-income-generating activities, such as leisure or childcare. However, 18% of their sample households place essentially zero monetary value on time savings from having water closer to their home. The remaining half of respondents (in two latent classes) valued time at roughly 25% of unskilled wages.

Cook et al.’s results are consistent with two quite different hypotheses about the value of time spent collecting water. First, water sector professionals have often speculated that women enjoy walking to collect water from outside the home because it gives them an opportunity to socialize with other women, and they enjoy getting out of the house. Second, others have argued that hauling water is hard, difficult work, and that water haulers should value a reduction in such grueling labor highly (Cook et al., 2018). Although a zero value of time savings could suggest that some water carriers may not mind collecting water outside the home, it might also mean that these households have few employment opportunities and are severely cash-constrained.

In summary, despite our warning above not to compare VOT estimates across sectors or activities, there is a surprising consistency in the VOT results in many of the studies summarized in Table 1. Nine of the eleven studies report mean estimates that fall in the range of 25%–75% of some measure of household income or wage rate. Only Kremer et al. (2011) report a mean value of (travel) time close to zero. And only Whittington et al. (1990) report a mean value close to the market wage rate of unskilled labor. There also appears to be no systematic differences in estimates derived from stated vs. revealed preference methods. We again note that nine of the eleven studies measured the value of time spent traveling (VTT).

## 3 Benefit transfer approach

The simplest approach to estimating the value of changes in time use in low- and middle-income countries is to assume that the average value of time of households affected by the health or development project being appraised is some fixed percentage of the average household’s wage rate. This “benefit transfer” approach effectively takes research findings from one location (the “study site”) and assumes that they will be applicable in another location (the “policy site”). This is in fact the most common approach in industrialized countries when the policy intervention affects households’ time use outside of their normal work day (i.e., “on their own time”).

In light of the findings from the admittedly limited literature on the value of time from developing countries reviewed in the previous section of this paper, we believe that the advice of Boardman et al. (2018) from industrialized countries is likely to be a good starting point for valuing changes in time use in developing countries. In other words, as a first approximation, in developing countries, households’ changes in time use outside of the formal sector can be estimated at 50% of the average after-tax wage rate. There is, however, an added complication in the application of this benefit transfer approach in developing countries, i.e., what is the after-tax wage rate in the policy site?

In industrialized countries, analysts can generally obtain sufficiently accurate data on local after-tax wage rates from government statistics. Although local wage or income data from representative national surveys are becoming more accessible in many low- and middle-income countries, they remain scarce in many locations. Analysts in developing countries may need to conduct some primary research on local after-tax wage rates in order to implement this benefit transfer approach. It is important to emphasize that the recommendation to value changes in time use at 50% of the after-tax wage rate does not mean that the analyst can substitute a conversion of annual (national) gross domestic product (GDP) per capita into an hourly estimate for the household’s after-tax wage rate, however convenient this substitution may seem. National GDP per capita (converted to $ per hour) is unlikely to be a close approximation of the average after-tax household wage rate in a specific location.

To obtain the average household after-tax wage rate, the analyst has three main options when secondary data are not available. First, the analyst can try to obtain self-reported household wages from a household survey like the Living Standards Measurement Survey (LSMS) and in some cases use information on tax withholding to calculate after-tax wages.^7^ Second, the analyst can collect self-reported income and convert this to an after-tax wage rate. Third, the analyst can gather information on the wage rate of unskilled labor in the policy site, and assume that this is the opportunity cost of labor to the average household.^8^

Recall that Boardman et al. (2018) also recommend using higher opportunity costs for time spent waiting or walking, reflecting the higher disutility most people in industrialized countries assign to spending time in those activities. Although it seems likely to us that an aversion to waiting is universal in human nature, it is also plausible that households in some countries and cultures are more accustomed to waiting than the households in the industrialized-countries populations that have been studied in most empirical applications. This would imply that households in developing countries perhaps place a lower premium on reductions in waiting times than those in industrialized countries, though they may still value reductions in wait times more than reductions in travel time.

Unfortunately, none of the studies from low- and middle-income countries reviewed above addressed this issue, so our recommendation for now is to value changes in time spent waiting at the same rate as time spent traveling. Similarly, there is currently no evidence on whether these populations value reductions in time spent traveling by motorized transport differently than time spent walking. In fact, most of the studies reviewed either involved household members walking (i.e., to collect water) or choosing among only motorized transport options. Only Jeuland et al. (2010) observed households making decisions about whether to walk or take public transport to a vaccination site, but their data do not allow the estimation of differential VTTs by mode. In the absence of empirical evidence, we again recommend valuing walking time the same as time traveling by other modes.

Similarly, because the available literature from developing countries is too thin to say much about the value of time changes across different activities (e.g., walking to collect water vs. walking to collect firewood, or waiting for public services vs. waiting in traffic), we again do not recommend the use of different percentages of after-tax wages for different activities. Instead we suggest that the analyst undertake a sensitivity analysis to determine whether the results of the benefit-cost analysis of the development project change for VOT changes between 25% and 75% of the after-tax wage rate.

As a simple example, suppose one were interested in estimating the economic benefits of improving water supply for households currently traveling to collect water. Suppose the project would provide them with piped connections in their compound. The two most important components of benefits will be improved health (via improved water quality and quantity) and a reduction in time spent collecting water. Cook et al. (2016*b*) surveyed 387 households in rural Kenya and found that the median household spends 2.35 hours per day collecting water during the dry season. If the project reduces these collection times to zero, and if they are valued at 50% of the local unskilled wage rate of 35 Kenyan shillings (Ksh) per hour, the project would yield time-savings benefits of 41 Ksh per day, or ∼1230 Ksh per month (see Table 3 in Cook et al. (2016*b*) for more details and alternative calculations). The sensitivity analysis would include values from 620 Ksh per month (25% of unskilled wages) to 1850 Ksh per month (75% of unskilled wages).^9^

This policy context also raises the issue of whose time is being saved: water collection is sometimes done by children (Sorenson et al., 2011), as are other types of resource collection. The opportunity cost of time for children may be reduced educational attainment rather than lost wages. Valuing time at a fraction of the unskilled wage rate may be an underestimate of the discounted stream of higher future earnings from improved investment in the child’s human capital if households did not understand the ultimate consequences of assigning work to children. Despite anecdotal evidence that this be true in many areas with poor access to water supply, there are currently few high-quality studies linking resource collection and educational attainment. One exception is Nauges (2017), who find that reducing Ghanaian girls’ water collection times in half is associated with increasing school attendance by 7%. It is also plausible, however, that reducing collection times may have a more important impact on educational attainment through increased time spent at night doing homework, or having more energy during the school day. Many parents may accurately judge the tradeoff between assigning their child work that they would either need to do themselves (at an economic cost) or hire outside help. If so, the link typically assumed between a child’s opportunity cost and the adult wage rate remains relevant.

Unfortunately, we know of no study that attempts to estimate the value of time for children separately from adults. In the absence of evidence, we recommend using 50% of the adult value when an analyst has reason to believe that a project saves the time of children who might plausibly perform work that could have been done by parents or adults. Assigning a shadow value of time to infants clearly would be inappropriate, but the age at which children begin household chores, as well as the number of hours they devote to them, will vary widely within and among countries. As a guideline, we recommend not assigning a shadow value of time saved by pre-school aged children.

Finally, we note that this benefit transfer approach does not capture the possibility of heterogeneity in the value of changes in time use among households affected by the health or development project. In other words, an estimate of the total benefits of time savings based on this benefit transfer approach will not reveal the differences in the value of the benefits among different household groups. As a result, while the analyst may be able to estimate how the amount of time use changes across various income or other groups, this benefit transfer approach may limit the analyst’s ability to assess the distributional consequences of the development project.

## 4 Stated preference approach to estimating the value of changes in time use: primary research

If an analyst wishes to estimate households’ value of time use changes in a specific local context, rather than use a benefit transfer approach, there are two primary options. The first is to rely on a revealed preference approach that uses households’ actual decisions that involve tradeoffs between time and money.^10^ As discussed in Section 2, these have most commonly involved transportation mode choices in industrialized countries, although Jeuland et al. (2010) used a revealed preference approach for the decision to travel to be vaccinated in Beira, Mozambique. The main concern with this approach is its applicability in rural areas of low-income countries. Unless by chance a natural experiment occurs (like the mass vaccination in Jeuland et al., 2010), transportation mode choices for most households may be too limited for the analyst to infer values of time from those decisions. In some settings, one might be able to gather data on households who choose to walk vs. take an informal taxi or motorbike. However, unless one gathers information on repeated choices from each respondent (e.g., panel data), it will be difficult to estimate anything except a population-level average VTT. If individual-level estimates are desirable for use in an analysis of the distribution of benefits and costs of a development project, then a stated preference approach generally will be preferable and cost less to implement. As discussed above, another application of the revealed preference approach is observing households’ choice of water source when the distance from the home and cost varies. This approach, however, requires information on water sources *not* chosen by the household, which is not available without a customized (special purpose) survey effort.

**Figure 1:**
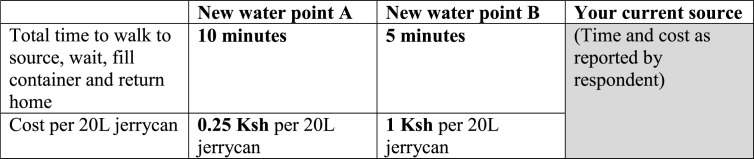
Hypothetical choice task in Cook et al. (2016*a*).

Although a stated preference approach for estimating the opportunity cost of time has been used in a number of studies in industrialized countries, particularly in transportation, we focus here on the recent approach in Cook et al. (2016*a*) since it was tailored for low- and middle-income countries.^11^ The basic tradeoff of time and money was framed in terms of water collection decisions. Respondents were first asked about the characteristics of the sources they currently use. They were then asked to imagine that two new *hypothetical* water source alternatives were available to them. They were told to assume that the characteristics of these new sources were very good (i.e., excellent and safe for drinking, open at convenient times), so that respondents could focus on changes in the two attributes that varied between the two hypothetical sources: (1) the price charged per 20-liter “jerrycan,” and (2) the time it would take to collect water from the source (including time waiting and filling the container). Using in-person interviews, the enumerator showed the respondent a printed choice task card (Figure 1) and explained the attributes associated with each hypothetical new water point (source), and asked if the respondent had any questions. Respondents were then asked which of the three water sources (their current main source and the two hypothetical sources) they would most and least prefer to use. The most and least-preferred data were used to construct a complete ranking of the two hypothetical choices and the respondent’s current primary source, which allowed the authors to estimate logit models that included all three options as well as models that only compared the choice between the two hypothetical sources.^12^

Although one could have each respondent complete only one such task, it is most common to have them complete a series of choice tasks, creating a panel of hypothetical choices that can then be used to model heterogeneity in values and estimate individual-level coefficients. The process of constructing these choice tasks is not simple, and is the domain of experimental design (see Hensher et al. (2015), Chapter 6 for an introduction). In the case of Cook et al. (2016*a*), the experiment was based on a “full factorial” design of two attributes (per jerrycan price and roundtrip time), each of which had three levels. Choice tasks where either hypothetical alternative dominated the other on both time and cost were eliminated, leaving nine choice tasks. These were then divided into three blocks with three choice tasks each. Respondents were randomly assigned to blocks, and task order within the block was randomized. In addition to the three tasks from the block, all respondents were presented with a task that included one source with the lowest time and lowest price and another source with the middle time and middle price. Because one of the two hypothetical sources dominated the other in both time and price, this task served as a simple check of respondents’ comprehension of the choice experiment.

An analyst could easily adapt this overall choice experiment structure to the most salient time–money tradeoff in their “policy site.” The water collection decision is likely to be relevant in many rural areas of low-income countries, especially in areas where respondents are familiar with paying per-trip or per-bucket fees. A similar tradeoff that is common in many rural areas is the decision to purchase charcoal vs. spend time collecting firewood. In urban areas or in middle-income countries, one could often use repeated hypothetical choice tasks focused on transportation decisions. For more information on conducting stated preference studies in low-income settings, interested readers might consult Whittington (2002) for discussions of scenario design, enumerator training and supervision, and study administration.

## 5 Recommendations

Perhaps surprisingly, we find that economists’ standard advice for valuing changes in time use outside of the formal sector in industrialized countries (i.e., use 50% of the household’s after-tax wage rate) seems to be applicable as well in low- and middle-income countries. However, because there are comparatively fewer studies and large, representative surveys of time use are rare, there remains considerable uncertainty in estimates of both time use and the shadow value of time in low- and middle-income countries. At the same time, a simple stated preference approach can be used for estimating the value of time changes specific to a “policy site.” This stated preference approach for valuing time changes has been tested in a developing country context (rural Kenya), and we believe that the results are encouraging.

We recommend that an analyst who is conducting a benefit-cost analysis of a development project in a developing country that results in significant changes in households’ time use first investigate to see if the majority of time changes are being devoted to income-generating activities outside the formal sector. If this is the case, then one should use the average household after-tax (i.e., “take home”) wage rate as the value of time. If data on average tax levels or deductions are unavailable, researchers can use gross wages or a local prevailing unskilled wage rate, though this should be clearly noted in the analysis. Time saved on the job that a worker can devote to other tasks for her employer should be valued at the before-tax wage rate plus benefits, although we have not addressed the potential difficulties in calculating the benefit packages of salaried employees in low- and middle-income countries.

If most of the time savings do not seem to be reallocated to income-generating activities, then the analyst should test whether an assumption of the value of time between 25% and 75% of the average after-tax wage rate in the policy site affects the outcome of the appraisal. We do not believe there is sufficient empirical evidence to use different values for different activities (i.e., waiting vs. walking). We also recommend using 50% of the adult value when an analyst has reason to believe that a project saves the time of children who might plausibly perform work that could have been done by adults. We recommend not assigning a shadow value of time saved by infants and pre-school aged children. If values of time within this range of 25%–75% do not affect the recommendation on the project, then primary research on the value of time in the policy site is probably not warranted. Alternatively, the analyst might conduct a break-even analysis of the opportunity cost of time needed for a project to pass a benefit-cost test. If this break-even value (expressed as a fraction of after-tax wages) is outside this 25%–75% range, more research on the VOT would be unlikely to affect the outcome of the benefit-cost analysis of the development project. In this case, primary research is again likely not warranted.

If the recommendation on the project (policy intervention) does change depending on the percentage of the after-tax household wage rate (between 25% and 75%), then the analyst should consider carefully the option of conducting primary research to estimate the monetary value households in the policy site impute to the activity-specific time changes. The stated preference approach described in Section 4 will likely be the main option that analysts have at their disposal to conduct such primary research.

There is one important caveat to this recommendation. If the distributional consequences of the policy intervention (e.g., the distribution of project benefits and costs on the poor or on women) are a central focus of the project, then primary research on the value of time changes may be justified even if the benefit-cost recommendation on the project does not change depending on the percentage of the after-tax household wage rate used to estimate the VOT. The benefit transfer approach will not reveal the heterogeneity among households in the policy site regarding their value of time. If understanding this heterogeneity among households is important to the analyst and her client, then there is little choice but to conduct primary research on the value of time changes in the policy site.

## Footnotes

1 As background for this paper, we reviewed all eight of the guidance documents cited in the Robinson et al. (2017) Scoping Study (see pp. 37–38). The documents vary in whether they focus specifically on the VTT or on other types of changes in the time needed for daily activities. In four cases, the guidance documents did not make recommendations on how to value time (Commonwealth of Australia, 2006; Treasury Board of Canada, 2007; US EPA, 2010; European Commission, 2015). Of these, some did not discuss valuing time at all (Canada and EU) while others discussed travel cost methods and valuations broadly without making specific recommendations (EPA (pp. 7.24–7.28) and Australia pp. 122–123). Two documents recommended valuing time lost at work at the employers’ labor costs (pretax wages plus benefits) and time lost from private time or leisure at 100% of post-tax wages (US HHS (2016) (pp. 26–32), Norwegian Ministry of Finance (2012) (pp. 41–48)). Both the UK and France recommend using specific values (in pounds and euros) for time savings in BCAs of transport policies (HM Treasury (2011) (pp. 59–60) and French Policy Planning Commission (2013) (pp. 33–35)). In France, these values are differentiated between urban and inter-urban transport, trip purpose, distance, mode (bus/train/auto), and activity (walking, waiting, transferring). Interestingly, three of these four documents that do make recommendations cite general economic theory rather than empirical work to support these rules around valuing changes in non-work and/or leisure time. The French and UK guidance document cite no empirical studies; US HHS (2016) refer readers to the Boardman et al. (2011) text and a US Department of Transportation report for further information. Finally, an older guidance document from the World Bank (Gwilliam, 1997) recommended valuing commuting and other non-work time for adults and children at 30% and 15% of hourly household income.

2 This is the type of tradeoff assumed in the model presented by Becker (1965).

3 Interestingly, a parallel convention arose in the recreational demand literature to value travel time to sites at 33% of wages (Phaneuf & Requate, 2016), based largely on early revealed preference empirical approaches (Cesario, 1976). This would be consistent with the idea that traveling *for recreation* would have less disutility than typical commuting travel. However, other studies report differing results. For example, Fezzi et al. (2014) use a rich dataset on beachgoers’ decisions of whether or not to use toll roads to access beaches on the Italian Riviera. They find that the value of time spent traveling to the beach is three-quarters of the average before-tax wage rate, but with large unobserved individual-level heterogeneity.

4 IT Transport (2005) used a stated preference approach, asking respondents about transportation mode choices in Bangladesh, Ghana and Tanzania. They find that the value of in-vehicle time is 64% of average hourly wages (without any adjustments for taxes) in Ghana, 49% in Tanzania and 51% in Bangladesh. They also tested whether WTP varied by comfort of the trip, quality of the road, income of the respondent, and the respondent’s ownership of a watch or time piece. They also interviewed a small number of children directly with the same set of questions. We do not include this study in Table 1 because several elements of the research design and stated preference methodology are not clearly reported, and the study does not seem to have undergone peer review.

5 Mas and Pallais (2017) implement a similar approach among 7,000 job applicants in the United States to explore the compensating wage differentials for schedule flexibility, working from home, and the ability of employers to change schedules on short notice. Workers were willing to accept wage offers 8% lower in exchange for being able to work from home, and 20% lower wages for jobs where the schedule was not set by the employer.

6 Kremer et al. (2011) observed only bounded values of time and money and assigned individual values based on the median of a normal distribution fit to the data (see footnote 18, page 185 of their paper). The underlying individual-level estimates are not reported or explored (only the summary statistics of the distribution of VTT are reported). To produce VTT estimates for the remainder of their sample, the paper mentions (page 185) a regression of these VTT estimates on education, number of children and asset ownership, but the results from this regression are not discussed or presented in the paper or in either of the two supplementary appendices.

7 In many but not all surveys the LSMS asked about taxes paid or withheld (i.e., Uganda 2011 or Tanzania 2010), so after-tax wages can be computed. In general, since the informal sector is larger in low- and middle-income countries and tax compliance may be lower, data on gross vs. take-home wages may be difficult to obtain. At the same time, however, the difference between the gross and after-tax measures is likely to be less important than in higher-tax industrialized countries.

8 When demand for labor is very low, e.g., during periods when little labor is needed in agriculture, it is not clear that even if the analyst had a wage rate, that it would be the right opportunity cost to use. For example, an individual might have a skilled job that pays 100 Ksh/hour, but the job is only available 8 hours per week. She cannot increase hours worked and earn 100 Ksh/hour if more time becomes available. If time “savings” are negative, then 100 Ksh/hour might be the appropriate shadow price because she would need to cut back on hours worked. But where households cannot increase labor supply even if they want to do so, labor market decisions are not in equilibrium. Feather and Shaw (1999) develop a framework to characterize different values of time depending on the person’s labor market situation (unemployed by choice, under-, over- or optimally employed). They ask respondents in a recreational demand study in the US questions designed to help classify them into these categories, and estimate class-specific values. In our context, one could in theory weight the opportunity cost of time by the probability of employment, but we know of no empirical applications that have attempted to do this. Using the unskilled wage rate seems more conservative and is plausibly a better estimate of the opportunity cost of time if at the margin someone with a little additional time available could find some work on activities requiring only unskilled labor.

9 1 US$ = 86 Ksh at the time of the study.

10 For introductions to these nonmarket valuation methods, we recommend Phaneuf and Requate (2016) and Haab and McConnell (2002).

11 Readers interested more broadly in stated preference methods should consult the recently updated guidance on conducting stated preference studies by Johnston et al. (2017).

12 This latter approach is important since the current water source may have unobserved attributes that provide utility or disutility, and respondents may anchor on their current status quo. Although one typically models such situations by including an alternate-specific constant (ASC), it can be useful to “strip out” that unobserved utility by comparing just the hypothetical alternatives in order to focus only on the marginal utility of time vs. the marginal utility of money. Note, however, that this latter model would likely do a poor job of predicting actual choices.
